# How to evaluate surgical tourism service organizations in China: indicators system development and a pilot application

**DOI:** 10.1186/s41256-022-00262-2

**Published:** 2022-08-16

**Authors:** Dan Zhang, Yue Yan, Mei-xia Liao, Ting-fang Liu

**Affiliations:** 1 Institute for Hospital Management, Tsinghua Shenzhen International Graduate School, Tsinghua University, No.2279 Lishui Road, Nanshan District, Shenzhen, 518000 People’s Republic of China; 2 School of Medicine, Tsinghua University, Beijing, 100084 People’s Republic of China; 3 Saw Swee Hock School of Public Health, National University of Singapore, Singapore, 119077 Singapore; 4 School of Health Management, Chinese Academy of Medical Sciences & Peking Union Medical College, Beijing, 100730 People’s Republic of China

**Keywords:** Surgical tourism, Evaluation system, Access criteria, Safety and quality

## Abstract

**Background:**

Surgical tourism is an emerging economic sector, with the most growth potential demonstrated in China's health industry before the COVID-19 pandemic. Surgical tourism accounts for a large part of medical tourism services in China, with high requirements in terms of quality and safety. By contrast, China suffers from insufficient measurement tools and theoretical research. The aim of this study was to develop a set of reliable and feasible indicators by augmenting the Donabedian model to evaluate the quality of surgical tourism services.

**Methods:**

A literature review and focus group interview were used to generate indicators for the quality of surgical tourism services. The basic framework of the evaluation system was based on the structure–process–outcome Donabedian model. The screening and weight setting were conducted through an analytical hierarchy process (AHP) and a two-round Delphi consultation with 13 panelists. The validity and reliability of experts were tested by the experts' positive coefficient, authority coefficient, and coordination coefficient. The reliability of the questionnaire was assessed by a pre-test distributed within an International Medical Department of a public hospital in China.

**Results:**

Based on the Donabedian quality theory, a novel evaluation system of surgical tourism service institutions was constructed with three dimensions, nine first-level items and 39 second-level items. The three dimensions consisted of the structure (0.315), process (0.287), and outcome (0.398), with several indicators for each dimension and each indicator was given a weight. Of the two rounds of Delphi consultation, the response rates were 86.67% and 100%. The coordination coefficient of expert opinions in the two rounds of consultation were 0.49 and 0.65 (*p* < 0.05). For the empirical study, the self-evaluation score of a public hospital was 86, which could rate as a two-star institution.

**Conclusions:**

Our evaluation system identified three suitable quality dimensions of surgical tourism services to improve the safety and quality of practical healthcare. It reflects the access criterion of surgical tourism institutions, provides references for the best choice of surgical services for tourists, and can be applied by healthcare managers and policy makers to allocate resources more efficiently and promote more surgical tourism services with international standards.

**Supplementary Information:**

The online version contains supplementary material available at 10.1186/s41256-022-00262-2.

## Introduction

Surgical tourism has become a new industry with the most growth potential demonstrated before the COVID-19 pandemic. The current global health and medical tourism market has been valued at about 60 billion US dollars, and the annual market consumption at about 21 billion US dollars, with an annual growth rate of 20–30% [1]. However, in the face of the pandemic crisis, the figure may decline. Compared with traditional tourism projects, surgical tourism tourists tend to stay in the area for long periods, involving higher medical consumption, which could effectively promote the development of hospitals, hotels, translation services, transportation, tourist attractions, shopping, and other related economic sectors. Surgical tourism has thus gradually become a new important aspect of the tourism economy, by combining people's demand for healthcare and their pursuit of leisure and entertainment [2–4].

Surgical tourism institutions provide medical services that are mainly aimed at physical examinations and surgical treatment such as cardiac diseases, cancers, and diabetes mellitus [5], which need to meet high safety standards. Surgical tourism is a sub-category of medical tourism, often involving long-distance travel for the specific surgical procedures [6]. Surgical tourism institutions providing surgical services have higher requirements in terms of safety and quality.

Due to the late start of China's surgical tourism, the surgical tourism market is in its initial phase, with major problems of inconsistency, and a lack of safety in terms of quality of care. At the same time, surgical tourism involves many departments such as medical treatment, tourism, transportation, exit and entry management, and thus decentralized management, which affects the sustainable development of China's surgical tourism industry.

Different treatments in Asian countries depend not only on standards and accreditations, but also on the patients' active mobility between continents and nations. For example, tourism activities for the patient’s companions and extra support for patients’ comfort have been identified as the important success factors of Korean surgical tourism. This thus suggests that in order to attract patient flow, the surgical tourism industry should support the patient and their companions to stay in a comfortable and pleasant environment [7, 8].

This study therefore focuses on surgical tourism institutions that have a high demand for medical treatments, and develops the access criterion and evaluation standards of surgical tourism service institutions.

The Chinese have long been an important source country for international medical tourism. In 2017, about 600,000 Chinese medical tourists went abroad, with an average spending of 7700 dollars per person, which was about 10 times that of ordinary tourists.

China is a big country with various tourism resources, and rich medical and surgical resources featuring traditional Chinese medicine. Faced with the continuous growth of the world’s surgical tourism market, how to standardize and develop China's surgical tourism industry has become of increasing interest in China and the world. This study focused on tourism and healthcare management and market competitiveness linked to surgical tourism, management tools, and future trends [9]. Asia has become one of the world's most potential surgical tourism markets, with Thailand, India, Malaysia, and Singapore representing big global players [10].

The Healthy China 2030 Plan issued in 2016 clearly stated that the standards and norms of the medical tourism industry should be formulated, competitive international medical tourism destinations should be created, and the integrated development of tourism and the health industry should be accelerated. In 2017, according to the Guidelines on Promoting the Development of Medical Tourism, the Chinese government set up 13 demonstration bases, represented by the Boao in Hainan Province as a Medical Tourism Pilot Zone. Encouraged by policies, many varied surgical service institutions have emerged. However, due to the lack of relevant access and evaluation standards, the services of the surgical tourism service institutions are fragmented, and thus the quality is uneven, which weakens the reputation of China's surgical tourism.

Research on the evaluation of surgical tourism service institutions at home and abroad was reduced by the COVID-19 pandemic and restricted traffic. In terms of research content, the existing studies mainly focus on the evaluation status of surgical tourism institutions, the theoretical framework of quality standards, and the service quality indicator system, etc. An evaluation indicators system can improve the quality of services provided by surgical tourism institutions, however surgical tourism has not been studied in much depth. In terms of research methods, current studies mainly use qualitative descriptions and analyses, while few use quantitative analyses and other methods [2–4]. The focus of this study was thus on the evaluation indicators system of surgical tourism service institutions, including the structure, process and outcome quality.

## Methods

### Research design

This study adopted mixed methods. A literature analysis of studies carried out in China and abroad was carried out, and the evaluation system was based on the Donabedian’s quality theory of structure quality, process quality and outcome quality. The empirical data were extracted from an International medical department of a public hospital in China through self-evaluation. The International Medical Department of the hospital selected in this study is the earliest foreign-related medical service institution in Beijing, and belongs to a tertiary A-level hospital. It has more than 40 clinical departments, including surgery, orthopedics, obstetrics, gynecology, pediatrics, and stomatology.

### Data collection and processing

This study collected evaluation indicators through a literature analysis, and extracted relevant evaluation items by referring to tourism industry standards. CNKI, Wanfang Data, and CQVIP were used to collect Chinese papers. The Springer Journal Database, SCI (Science Citation Index) Database and Wiley Online Library were used to collect English-language papers. The search terms included surgical tourism services, market access criterion, and evaluation indicators. Irrelevant papers were removed by browsing the abstract. The literature with a high correlation was classified and managed, and finally 13 related studies were obtained. In addition, evaluation indicators were also extracted by referring to standard documents such as Hainan Province Health Tourism Base Construction Standard and National standard GB/T17775-2003.

The two-round Delphi method was used to screen the evaluation indicators and determine the evaluation indicator system. According to the primary indicators, we designed an Expert Consultation Table. A total of 15 front-line experts from universities, medical institutions and industry associations who had worked in the relevant fields for long periods were invited for consultation, and the questionnaire was emailed to them. The reliability of the experts was verified by an empirical case research. The data were input and sorted by IBM SPSS Statistics 23.0 and Excel software. The indicators were organized into a three-part questionnaire, which was pre-tested and distributed within an International medical department of a public hospital in China, to assess its reliability and validity.

### Analysis and rating

The Analytic Hierarchy Process (AHP) was used to analyze all kinds of indicators and determine the weight of each indicator. The AHP is a structured technique for organizing and analyzing decisions, based on mathematics and psychology. It represents an accurate approach to quantifying the weights of decision criteria. Individual experts’ experiences are used to estimate the relative magnitudes of factors through pair-wise comparisons. Each of the respondents compares the relative importance of each pair of items using a specially designed questionnaire [11]. On the basis of the selected indicators, the questionnaire on the Evaluation Indicator Weight Assignment of Surgical Tourism Service Organizations was formulated, and a total of 13 experts were invited to fill it in. All the questionnaires passed the consistency test, and the geometric average method was then used to synthesize the average to obtain the final group judgment matrix. The AHP was calculated and analyzed using Expert Choice 2007, an AHP analysis software.

After the evaluation, the grades of the different surgical tourism service institutions were classified by a star rating. Only when institutions met all the corresponding standards could it be awarded the corresponding star rating. The scoring criteria of each of the star surgical tourism institutions are as follows: only if the total score of surgical tourism institution exceeds 60 points, it can be rated as passed; If the total score exceeds 70 points, it can be rated as a 1-star surgical tourism institution; if the total score exceeds 80 points, it can be rated as a 2-star surgical tourism institution; if the total score exceeds 90 points and all core indicators meet the standards, it can be rated as a 3-star surgical tourism institution.

## Results

### The preliminary indicators system

Evaluation indicators were collected and sorted to form an indicator database, the nine first-level items of which were: the structure of the organization, the institutional improvement, and service assurance in the structure; the operations management, service supervision, and service project in the process dimension, and service efficiency and effectiveness, and discipline development and influence on the outcome. The evaluation indicators were preliminarily screened according to the principles of reliability, timeliness, systematisms and hierarchy [12–15].

### Indicators screening and indicators system construction

#### Consultation with experts

In this study, 15 experts from different fields were selected for the Delphi expert consultation, and 13 completed questionnaires were collected. A total of 10 (76.9%), experts were mainly employed in hospital management, 2 (15.4%) in clinical diagnosis and treatment, and 1 (7.7%) in scientific research. A total of 10 experts (76.9%) were professor. A total of 11 experts (84.6%) were from hospitals, with senior experience and research in hospital management. A total of 11 employees (84.6%) had been working in related fields for more than 10 years and had good working experience. The basic information on the experts is shown in Table 1.

**Table 1:** Basic information on experts

Items	Category	Frequency	Proportion (%)
Age	Under the age of 45	1	7.7
	Between 46 and 55	7	53.8
	Between 56 and 65	5	38.5
Education background	Master’s degree	6	46.2
	PhD degree	7	53.8
Main working field	Clinical diagnosis	2	15.4
	Hospital management	10	76.9
	Scientific research	1	7.7
Professional title	Associate professor	3	23.1
	Professor	10	76.9
Working organization	Hospital	11	84.6
	Universities/research institutions	1	7.7
	Industry	1	7.7
Years of work experience	Under 10 years	2	15.4
	Between 11 and 20	6	46.2
	Between 21 and 30	3	23.1
	More than 30 years	2	15.4
Time spent directly serving patients	Without or < 10%	5	38.5
	10–24%	4	30.8
	25–49%	2	15.4
	50–74%	1	7.7
	> 75%	1	7.7

#### Expert advice

According to the expert scores on the indicators, the boundary value method was adopted to screen the indicators according to the full marks rate, rank sum, weighted average, and coefficient of variation.

According to the first round of expert advice, the inclusion criteria of indicators were determined as follows: full mark rate > 0.65, weighted average > 8.37, coefficient of variation < 0.21. A total of one second-level indicator and 12 third-level indicators were removed. One second-level indicator, and four third-level indicators were added, whereas two second-level indicators were modified. Experts also divided all evaluation indicators into necessary indicators, core indicators and bonus indicators.

According to the second round of expert advice, the inclusion criteria were: full mark rate > 0.85, equal-weighted average > 8.51, coefficient of variation < 0.19. A total of 11 second-level indicators were then modified. In general, because the indicators system was targeted at surgical tourism institutions, experts believed that certain indicators could be stricter, such as the bed  to nurse ratio, proportion of senior professional doctors, patients' satisfaction, employee satisfaction, and social satisfaction indicator. However they believed that the technical error rate, and incidence of hospital-acquired infection should be set lower. In addition, for medical service center, medical equipment, and centralized construction, experts suggested that these should be determined according to the strategic positioning and business scope of the different institutions.

#### Final evaluation indicators system of surgical tourism service organizations

The evaluation system was further modified and the indicators were finally determined based on the experts' scores, modifications, and additional suggestions. The evaluation system consisted of 3 dimensions, including 9 first-level indicators, 39 second-level indicators, 27 necessary indicators, 12 bonus indicators. Necessary indicators are necessary conditions for surgical tourism service organizations, see Table 2 for details.

**Table 2:** Evaluation indicators system of surgical tourism service organizations

Dimension	I Level indicators	II Level indicators	Comment
1. Structure quality	1.1 Organization structuring	1.1.1 Qualifications and practices	Necessary item
		1.1.2 Cultural advancement	Necessary item
		1.1.3 Architecture and environment	Necessary item
		1.1.4 Organizational management structure	Necessary item
		1.1.5 Disease and specialty construction	Necessary item
		1.1.6 Surgical tourism service centre	Necessary item
		1.1.7 Location and surrounding environment	Necessary item
		1.1.8 Hospitalization service settings	
		1.1.9 Outpatient service setup	
	1.2 Institutional improvement	1.2.1 Service planning and positioning	Necessary item
		1.2.2 Rules and procedures	Necessary item
	1.3 Service assurance	1.3.1 Staffing basics	Necessary item
		1.3.2 Infrastructure and equipment	Necessary item
		1.3.3 Hospital environment	Necessary item
		1.3.4 Medical service center	
		1.3.5 Medical equipment	
		1.3.6 Logistic support service	
2. Process quality	2.1 Operations management	2.1.1 Management and certification	Necessary item
		2.1.2 Capacity building	Necessary item
		2.1.3 Emergency and complaint response	Necessary item
		2.1.4 Information construction	Necessary item
		2.1.5 Marketing and publicity	
	2.2 Service supervision	2.2.1 Core healthcare systems and patient safety goals	Necessary item
		2.2.2 Personal privacy and health records management	Necessary item
		2.2.3 Dispute prevention and settlement	Necessary item
		2.2.4 Infection control	Necessary item
		2.2.5 Medical ethics management	Necessary item
		2.2.6 Continuity of service	
	2.3 Service project	2.3.1 Multiplicity of service	Necessary item
		2.3.2 Prices and charges	Necessary item
		2.3.3 Personalized service	
		2.3.4 Other services	
3. Outcome quality	3.1 Service effectiveness	3.1.1 Performance and safety	Necessary item
		3.1.2 Satisfaction	Necessary item
	3.2 Service efficiency and effectiveness	3.2.1 Efficiency of surgical tourism services	Necessary item
		3.2.2 Economic effectiveness	Necessary item
		3.2.3 Awards	
	3.3 Discipline development and influence	3.3.1 Academic impact and achievements	
		3.3.2 Teaching and training	

#### Expert positive coefficient, authority coefficient and coordination coefficient

Expert positive coefficient: It is generally believed that the questionnaire recovery rate of Delphi method reaches more than 70%, which means that the expert is highly positive coefficient. The first round issued 15 consult tables, with 13 returned, all of which were valid, with a recovery rate of 86.67%. The second round issued 13 consult tables, with 13 copies returned back, and a 100% recovery rate. The results showed that the Delphi expert positive coefficient was high, which also means that 13 Delphi consulting experts were interested in this research.

Expert authority coefficient: This study adopted a self-evaluation method, and considered the authority of experts in terms of their familiarity with the issues represented by the indicators (CS) and the judgment basis for expert evaluation of the indicators (CA), which was reflected by the authority coefficient (CR), in which: Cr = $\frac{Cs+Ca}{2}$, 0 < Cr < 1. The results showed that the average familiarity coefficient (CS) of each key indicator of the two round consultation was 0.83 and 0.86, respectively. This indicates that the experts were familiar with this topic. The average value of the CA was 0.78 and 0.80 respectively, indicating that CA had a high influence on the experts. The average expert authority coefficient (CR) of the two round consultation was 0.81 and 0.83 respectively, which indicates that the authority of the experts was high.

The expert coordination coefficient reflects the consistency of the evaluation of each indicator among different experts and can also be used as an indicator to reflect the credibility of the expert consultation. The coordination coefficient of the expert opinions in the first round of consultation was 0.49. Some experts had different opinions on the importance of the evaluation indicators, and the overall degree of coordinated opinions was low. The result of the coordination coefficient in the second round of consultation was 0.65, which was greatly improved compared with the first round, indicating that the experts' understanding of the importance of the indicators ì gradually converged, and the coordinated degree of expert opinions was relatively high. The χ^2^ test *P* values of the two rounds of coordination coefficient were all less than 0.05, indicating that the result had a 95% confidence level.

### Evaluation method and process

#### Weight setting

A total of 13 expert questionnaires were sent out for the indicator weight assignment survey, with a return rate of 100%. The effective rate of the tested questionnaires was 100%. See Table 1 for the personal data of the experts. The judgment values of 13 expert questionnaires were input into the statistical software, and all 13 questionnaires passed the consistency test. The geometric average method was used to integrate the average to obtain the final group judgment matrix. The consistency ratio of the model in this study was less than 0.1, indicating that the judgment matrix had a satisfactory consistency, and the weight of each factor calculated was credible. The software was used to analyze and calculate the weight of each item, and the final weight is shown in Table 3. Among them, more than 3 scores are core indicators.

**Table 3:** Evaluation indicator weight and score table of surgical tourism service organizations

Evaluation dimensions	First level indicators	Second level indicators	Weight	score	Evaluation method
1. Structure quality (0.315)	1.1 Organization structuring (0.095)	1.1.1 Qualifications and practices	0.018	2	Literature
		1.1.2 Cultural advancement	0.005	0.5	Literature
		1.1.3 Architecture and environment	0.007	0.5	Site rating
		1.1.4 Organizational management structure	0.012	1	Literature
		1.1.5 Disease and specialty construction	0.004	0.5	Literature
		1.1.6 Surgical tourism service center	0.007	1	Site rating
		1.1.7 Location and surrounding environment	0.012	1	Site rating
		1.1.8 Hospitalization service settings	0.015	1.5	Site rating
		1.1.9 Outpatient service setup	0.015	1.5	Site rating
	1.2 Institutional improvement (0.123)	1.2.1 Service planning and positioning*	0.041	4	Literature
		1.2.2 Rules and procedures*	0.082	8	Literature
	1.3 Service assurance (0.097)	1.3.1 Staffing basics	0.028	2.5	Literature
		1.3.2 Infrastructure and equipment	0.013	1.5	Literature
		1.3.3 Hospital environments	0.013	1.5	Site rating
		1.3.4 Medical service center	0.023	2.5	Site rating
		1.3.5 Medical equipment	0.007	0.5	Site rating
		1.3.6 Logistics support service	0.013	1.5	Site rating
2. Process quality (0.287)	2.1 Operations management (0.128)	2.1.1 Management and certification	0.013	2	Literature
		2.1.2 Capacity building	0.033	3	Literature
		2.1.3 Emergency and complaint response	0.021	2	Literature
		2.1.4 Information construction*	0.053	5	Site rating
		2.1.5 Marketing and publicity	0.008	1	Literature
	2.2 Financial supervision (0.118)	2.2.1 Core healthcare system and patient safety goals	0.031	3	Data acquisition literature
		2.2.2 Personal privacy and health records management	0.022	2	Site rating
		2.2.3 Dispute prevention and settlement	0.022	2	Site rating
		2.2.4 Infection control	0.031	3	Site rating
		2.2.5 Medical ethics management	0.006	0.5	Literature
		2.2.6 Continuity of service	0.006	0.5	Literature
	2.3 Service project (0.041)	2.3.1 Multiplicity of services	0.007	1	Site rating
		2.3.2 Prices and charges	0.007	1	Site rating
		2.3.3 Personalized service	0.025	2.5	Site rating
		2.3.4 Other services	0.002	0.5	Site rating
3. Outcome quality (0.398)	3.1 Service effectiveness (0.22)	3.1.1 Performance and safety*	0.108	11	Literature Data acquisition
		3.1.2 Satisfaction*	0.112	11	Data acquisition
	3.2 Service efficiency and effectiveness (0.123)	3.2.1 Efficiency of surgical tourism services*	0.062	6.5	Data acquisition
		3.2.2 Economic effectiveness*	0.051	5.5	Data acquisition
		3.2.3 Awards	0.010	0.5	Literature
	3.3 Discipline development and influence (0.055)	3.3.1 Academic impact and achievements	0.023	2	Data acquisition
		3.3.2 Teaching and training	0.032	3	Literature

* denotes core indicator, there are 7 core indicators in total

#### Evaluation procedure

In terms of the review process, this study divided the evaluation process into the early evaluation period, the mid-term evaluation period, and the late evaluation period. In the early stage, the surgical tourism institution carries out a self-evaluation according to the criteria and reports it to the public. During the evaluation period, the agency in charge of the evaluation classifies, numbers and registers the relevant materials of the surgical tourism institution, and carries out a document review and on-site evaluation. In addition, the agency submits a star rating evaluation, writes the evaluation reports, and gives feedback of the evaluation results to relevant organizations and the evaluated surgical tourism organizations. In the later stage of evaluation, the evaluation results are published, and the evaluated surgical tourism institution makes continuous improvements according to the recommendations put forward in the evaluation report. Four years after this period, a new round of evaluation is carried out [16–20].

#### Pilot application

The constructed indicators system and the evaluation procedure of the surgical tourism service institutions carried out in this study were empirically piloted in an International Medical Department of a public hospital in China through a self-evaluation. The institution's overall score was 86, which was made up of the structure–process–outcome dimensions scores of 31.5, 29.0, 39.5 points respectively, which would earn them a two-star rating, the final scoring results are shown in Additional file 1. The hospital performed well in some aspects. The second-level items with full marks were qualifications and practices, cultural advancement, disease and specialty construction, and so on. However, in addition to the above plus points, the hospital had a few additional services that needed to be improved. The empirical study proved that the indicators system constructed in this study was reliable and operable, however it still needs to be evaluated and perfected in other Chinese surgical medical institutions.

## Discussion

### Features of the indicators system

The quality connotation and safety of surgical services is the core of the surgical tourism evaluation. The evaluation system attempted to ensure that surgical tourism institutions meet basic safety standards and have appropriate medical equipment to perform the high-quality procedures offered. This study therefore used the Donabedian quality evaluation model as a reference and constructed the evaluation indicators system of surgical service institutions from the three dimensions of structure, process, and outcome.

The structural dimension focuses on the institutional setup, and service guarantee, the process dimension focuses on the operations, service supervision and service projects, and the outcome includes the evaluation of the service efficiency, effects and benefits, discipline development and influence. After two rounds of expert consultation, the evaluation indicator constructed consisted of 3 dimensions, 9 first-level indicators, and 39 second-level indicators, including 27 necessary indicators (7 core indicators) and 12 bonus indicators. This indicators system includes both access indicators and development indicators.

The necessary and core indicators can be used as the access conditions for medical institutions to carry out surgical tourism services, and the bonus indicators indicate how future surgical tourism service institutions can make improvements. The comprehensive and diversified indicators were set up to meet the needs of multiple users, so that tourists, healthcare managers, policy makers can all benefit from the indicators [21].

The weight setting of the evaluation indicators system showed that in terms of structure, process and outcome, the weight of the outcome quality was highest followed by structure quality and process quality. This indicates that, as a surgical tourism service organization providing international high-quality medical services, it has high requirements in terms of service efficiency, effects, and benefits, with satisfaction, performance and safety being the key factors for the evaluation of the organization. In addition, perfect system construction, service guarantee and other structural factors are important to ensure the institutions provide high-quality surgical tourism services. This indicators system proved to be reliable and valid. Various institutions were divided into three grades by the star rating system. An empirical study was conducted on the international medical department of a hospital.

This research built the evaluation indicators system is the basic requirements for surgical tourism institutions, and trying to standardized the service behavior of surgical tourism services, to maintain the legitimate rights and interests of consumers, and to establish the surgical tourism services of fair competition market, added to China’s surgical tourism services evaluation class health research gaps.

### Strengths and limitations

The first significant advantage of this indicators system lies in the emphasis on surgical quality and safety. Compared with the evaluation indicators system of other tourism institutions, this one focuses more on quality and safety, the supporting services in terms of language, insurance, reimbursement, and so on. In addition, international patient practices need to be conformed with in terms of ensuring quality and safety, and respecting diverse cultures. Secondly, the indicators system highlights the unique advantages of China's surgical tourism. This study provides a reference for the comparability of service quality for traditional Chinese medicine (TCM) medical institutions [22]. It also highlights the need to create personalized services and a TCM tourism brand to attract foreign tourists [23].

In order to improve the practicability of the indicator system, an evaluation table was created, and institutions were evaluated by a percentage system. The evaluation indicators system could also help tourists to make better choices regarding suitable services, and can help healthcare managers highlight the market access criterion and gain better outcomes, and also help policy makers to allocate resources more efficiently and guarantee higher levels of surgical tourism services in China.

Several limitations need to be highlighted. First, the evaluation indicators system has only been used in one hospital. The applicability of the system needs further verification in other institutions. The next stage will involve large-scale empirical studies on different types of medical institutions for comparison. Second, the 2019 coronavirus outbreak had a major impact on the international surgical tourism due to the travel restrictions and border control measures [24]. Risk perception could be a significant factor for tourists’ decisions [25], and safety regulations and procedures become more important for organizations [26]: these regulations and procedures were not evaluated in the study. The behavior of travelers therefore merits further study.

### Applications

The evaluation indicators system of surgical tourism in this study showed a good validity and reliability and could be applied in various ways. For tourists in China, it could be used to make better choices regarding surgical tourism services and destinations, with the uniform service evaluation standards ensuring consistent quality. Tourists would not have to worry about where to buy the service, and whether they were getting value for money. It could help the public to better choose the surgical tourism service agencies suitable for their own needs.

Secondly, the evaluation indicators system could help organizations highlight their own strengths, weaknesses and make future improvements, guiding medical staff to improve service quality and increase cultural management skills [27]. The surgical tourism market access criterion also helps institutions to monitor themselves.

Finally, it could also help the government to promote the quality management and supervision of surgical tourism and surgical services [28]. In the future, more empirical studies are needed to promote the standardization of international surgical tourism service models [21]. The COVID‐19 pandemic has nearly frozen cross‐border surgical tourism. Future surgical travel may also be deterred by the combination of the uncertain public health response to the pandemic, further travel restrictions, vaccine coverage, and pandemic‐related disruptions among medical service providers [29].

## Conclusions

In this study, the Donabedian quality evaluation model was adopted to construct the evaluation indicators system of surgical tourism service institutions, including 3 dimensions, 9 first-level indicators, and 39 second-level indicators. Overall this led to a relatively comprehensive evaluation of surgical tourism service institutions. At the same time, this study also defined the indicator weight, evaluation tools, methods, and processes, with a certain degree of operability and generalization.

Travel needs to be aligned with political and economic factors, however this study plays a positive role in the development and supervision of surgical tourism service institutions, and is conducive to promoting China's surgical tourism service at international standards. Surgical travel is facing new forms of competition due to the long-term and short-term impact of the pandemic. For example, both Malaysia and South Korea are using telemedicine and other services to maintain a foothold in the global market. Integration with data information services is likely to be the focus of future trends.

## Supplementary Information

**Additional file 1.** Scoring results of the International Medical Department of a hospital.

## Data Availability

The datasets supporting the conclusions of this article are included within the article and its additional files. The data are available upon reasonable request, and so are not publicly available.
