# Supplementary material for: How to evaluate surgical tourism service organizations in China: indicators system development and a pilot application
Source: Glob Health Res Policy. 2022 Aug 16;7:26. doi: 10.1186/s41256-022-00262-2 (PMC9378254; doi:10.1186/s41256-022-00262-2)
Supplement: Supplementary file 1 — Additional file 1. Scoring results of the International Medical Department of a hospital. [file 41256_2022_262_MOESM1_ESM.docx]

Attachment 1 Scoring results of the International Medical Department of a hospital

| Evaluation dimensions | First level indicators | Second level indicators | Standard Score | Score |
| --- | --- | --- | --- | --- |
| 1. Structure quality | 1.1 Organization structuring | 1.1.1 Qualifications and practices | 2 | 2 |
|  |  | 1.1.2 Cultural advancement | 0.5 | 0.5 |
|  |  | 1.1.3 Architecture and environment | 0.5 | 0.4 |
|  |  | 1.1.4 Organizational management structure | 1 | 0.8 |
|  |  | 1.1.5Disease and specialty construction | 0.5 | 0.5 |
|  |  | 1.1.6 Surgical tourism service center | 1 | 0.5 |
|  |  | 1.1.7 Location and surrounding environment | 1 | 0.7 |
|  |  | 1.1.8 Hospitalization service settings | 1.5 | 1.3 |
|  |  | 1.1.9 Outpatient service setup | 1.5 | 1.3 |
|  | 1.2 Institutional improvement | 1.2.1 Service planning and positioning * | 4 | 3.7 |
|  |  | 1.2.2 Rules and procedures * | 8 | 7 |
|  | 1.3 Service assurance | 1.3.1 Staffing basics | 2.5 | 2 |
|  |  | 1.3.2 Infrastructure and equipment | 1.5 | 1.5 |
|  |  | 1.3.3 Hospital environments | 1.5 | 1.2 |
|  |  | 1.3.4 Medical service center | 2.5 | 2 |
|  |  | 1.3.5 Medical equipment | 0.5 | 0.5 |
|  |  | 1.3.6 Logistics support service | 1.5 | 1.1 |
| 2.Process Quality | 2.1 Operations Management | 2.1.1 Management and Certification | 2 | 2 |
|  |  | 2.1.2 Capacity building | 3 | 2.7 |
|  |  | 2.1.3 Emergency and complaint response | 2 | 1.7 |
|  |  | 2.1.4 Information construction* | 5 | 4.5 |
|  |  | 2.1.5 Marketing and publicity | 1 | 0.8 |
|  | 2.2 Financial Supervision | 2.2.1 Core healthcare system and patient safety goals | 3 | 3 |
|  |  | 2.2.2 Personal privacy and health records management | 2 | 1.7 |
|  |  | 2.2.3 Dispute prevention and settlement | 2 | 2 |
|  |  | 2.2.4 Infection control | 3 | 2.7 |
|  |  | 2.2.5 Medical ethics management | 0.5 | 0.4 |
|  |  | 2.2.6 Continuity of service | 0.5 | 0.5 |
|  | 2.3 Service Project | 2.3.1 Multiplicity of services | 1 | 0.7 |
|  |  | 2.3.2 Prices and charges | 1 | 1 |
|  |  | 2.3.3 Personalized service | 2.5 | 2 |
|  |  | 2.3.4 Other services | 0.5 | 0.2 |
| 3.Outcome Quality | 3.1 Service Effectiveness | 3.1.1 Performance and safety* | 11 | 9 |
|  |  | 3.1.2 Satisfaction* | 11 | 10 |
|  | 3.2 Service efficiency and effectiveness | 3.2.1 Efficiency of surgical tourism services* | 6.5 | 5.8 |
|  |  | 3.2.2 Economic effectiveness* | 5.5 | 5.3 |
|  |  | 3.2.3 Awards | 0.5 | 0.2 |
|  | 3.3 Discipline development and influence | 3.3.1 Academic impact and achievements | 2 | 1.3 |
|  |  | 3.3.2 Teaching and Training | 3 | 1.5 |
| Total score |  |  |  | 86 |
